# Cardiac lymphoma requiring urgent heart transplant due to ventricular tachycardia storm: a case report

**DOI:** 10.1093/ehjcr/ytaf279

**Published:** 2025-06-05

**Authors:** Julián Lugo-Peña, Héctor M Medina, Juan P Umaña, María Daniela Valderrama-Achury, Adriana Torres

**Affiliations:** Department of Clinical Cardiology, Fundación Cardioinfantil-Instituto de Cardiología, Calle 163A # 13B-60, Bogotá 110311, Colombia; Cardiac Imaging, Texas Heart Institute, 6770 Bertner Avenue, Houston, TX 77030, USA; Baylor College of Medicine, 1 Baylor Plz, Houston, TX 77030, USA; Chair, Division of Thoracic & Cardiovascular Surgery, Cleveland Clinic, 3100 Weston Road, Weston, FL 33331, USA; Research Institute of the McGill University Health Centre, 845 Sherbrooke Stw, Montreal, Quebec, Canada, H3A 0G4; Heart Failure Unit Los Cobos Medical Center, Avenida 9 " 131-40, Bogotá 111321, Colombia

**Keywords:** Cardiac lymphoma, Cardiac transplantation, Chemotherapy, Long-term follow-up, Ventricular tachycardia, Case report

## Abstract

**Background:**

Primary cardiac lymphoma (PCL) involves the heart almost exclusively although it can extend to surrounding structures including the pericardium. Most PCLs in adults are of B-cell origin and their signs and symptoms are generally non-specific and depend on their location and size. In general, cancer patients usually have a slim chance of receiving heart transplantation (OHT), although it’s not an absolute contraindication depending on the decision of the multidisciplinary team and the experience of each institution.

**Case Summary:**

A 48-year-old man, with an ultimate diagnosis of primary cardiac follicular B-cell lymphoma presented to our hospital mimicking hypertrophic cardiomyopathy. He initially presented with worsening heart failure and ventricular tachycardia storm (VT-S) that required urgent cardiac OHT. The final pathological analysis of the explanted heart revealed the presence of a PCL without extra-cardiac extension. In addition to initial immunosuppression with mycophenolate mophethyl, corticosteroids, and tacrolimus, he was switched to Everolimus and dose reduction of Tacrolimus. Rituximab + Bendamustine was initiated to reduce the risk of cardiotoxicity and myelotoxicity associated to R-CHOP. The follow-up body PET-CT, *trans*-thoracic echocardiogram, cardiac magnetic resonance imagings and biopsies were normal. During a regular follow-up heart biopsy procedure to ascertain rejection, the patient developed torrential tricuspid regurgitation and required surgical valve replacement.

**Discussion:**

After 5.5 years of follow-up, the patient remains asymptomatic, with normal graft function, in NYHA FC I, and without oncological relapses despite receiving a modified chemotherapy regimen. Selected patients with a PCL can be managed with OHT and a modified chemotherapy regimen. They could also be followed up using a non-invasive approach to monitor rejection.

Learning points

- Hypertrophic phenotype is a clinical and echocardiographic syndrome and accurate diagnosis is essential for optimal medical care.
- Patients with rapidly deteriorating heart failure, independent of LVEF, must be early referred to tertiary care centres to advance and expedite their workup and final treatment.
- Management of PCL is very complex and infrequently can be accomplished with a heart transplant and very close follow-up is needed due to the complex combination of anti-rejection medications and chemotherapy agents.

## Introduction

Primary cardiac lymphoma (PCL) involves the heart almost exclusively although it can extend to surrounding structures including the pericardium. In adults, most PCLs are of B-cell origin and their signs and symptoms are generally non-specific including clinical heart failure syndrome (ranging from mild to severe), pericardial effusion, embolic phenomena, and arrhythmias, depending on the location and the extent of the mass. PCL may be considered as part of the differential diagnosis in patients with increased end-diastolic thickness.^1–3^

## Case presentation

A 48-year-old immune-competent, Latin male, without prior medical history, presented to an outside hospital with a 7-month history of progressive dyspnoea on exertion, orthopnea, and non-cardiac chest pain. The patient presented with vital signs within normal limits. Pulmonary auscultation revealed bilateral crackles at the lung bases. Cardiovascular examination demonstrated jugular venous distension, and the lower extremities showed bilateral pitting oedema. The patient's complete blood count (CBC) and lactate dehydrogenase (LDH) levels were within normal limits. However, the high-sensitivity troponin I and NT-proBNP levels were elevated. The initial *trans*-thoracic echocardiogram (TTE) demonstrated normal left-ventricular systolic function with markedly increased left-ventricular end-diastolic thickness. Due to these clinical and echocardiographic findings, he was initially diagnosed with heart failure syndrome with preserved ejection fraction and was started on medical therapy including beta-blockers, angiotensin receptor antagonist, and a mineralocorticoid antagonist. His functional class and left ventricle ejection fraction (LVEF) deteriorated (from 50% to 35%) in the next few weeks requiring multiple clinical heart failure re-admissions. A single-photon emission computed tomography (SPECT) showed a perfusion defect suggesting ischaemia in the anterolateral wall that was followed by a normal coronary angiogram. He eventually had a sudden cardiac death (SCD) episode due to ventricular tachycardia (VT) which prompted an endomyocardial biopsy and insertion of an implantable cardiac defibrillator (ICD) and was ultimately transferred to our institution. At this point, differential diagnoses include hypertrophic cardiomyopathy, Fabry´s disease, and early-onset Amyloidosis.

On admission, he had multiple episodes of VT and atrial flutter treated with ICD therapies followed by epicardial and endomyocardial ablation.

**Figure 1:**
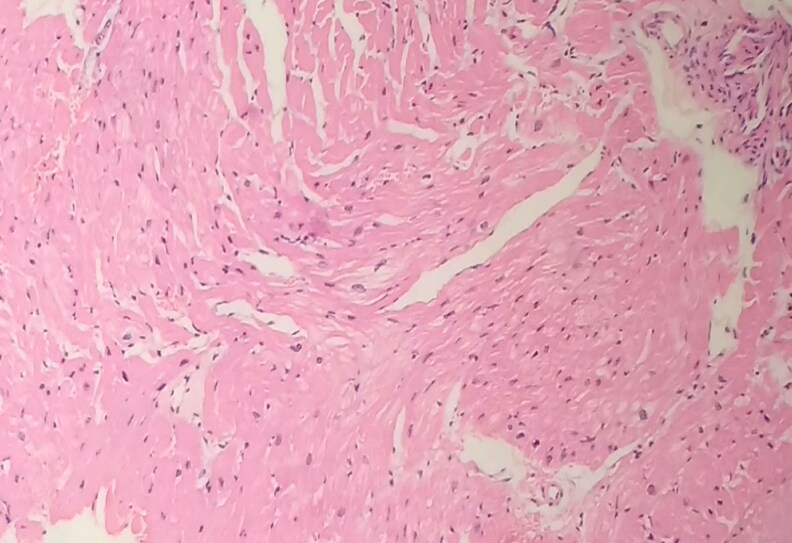
First instantaneous myocardial histology slice performed by percutaneous endomyocardial biopsy presenting only non-specific dilated cardiomyopathies changes.

A new TTE showed severe left-ventricular systolic dysfunction (LVEF 25%) and a marked increase in the left ventricle end-diastolic thickness (up to 25 mm with increased echogenicity) in the basal antero-septum. The gross pathology review of endomyocardial biopsies reported changes of hypertrophic myocardial fibres suggestive of hypertrophic cardiomyopathy and an alpha-galactosidase test ruled out Fabry's disease. (*Figure 1*).

**Figure 2:**
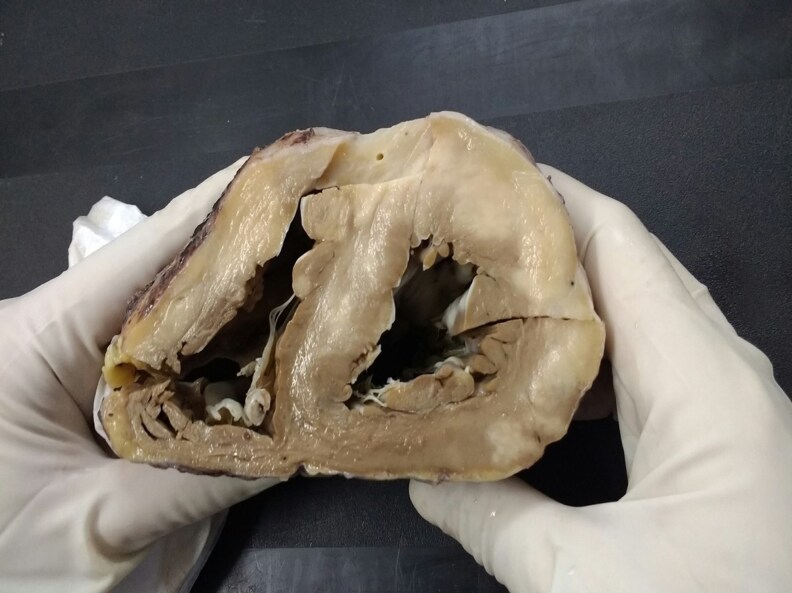
Macroscopic view of the surgical specimen showing thickened myocardial walls in both ventricles, with *raw-fish* aspect, respecting the endocardial border at the interventricular septum.

The patient’s condition worsened due to ventricular tachycardia storm (VT-S) despite maximal medical therapy. An emergency evaluation for inclusion in the heart transplantation list was performed and, due to his extremely critical condition, he was deemed as status 1A in the national waiting list. Two weeks later, an OHT was performed, and the explanted heart showed thickened myocardial walls in both ventricles, with a macro *raw-fish* aspect, respecting the endocardial border at the interventricular septum. (*Figure 2*).

The patient had an uneventful post-operative course and was started on Basiliximab induction per protocol, glucocorticoids, and mycophenolate mofetil (750 mg twice daily) followed by tacrolimus (adjusted to therapeutic levels), and anti-microbial prophylaxis.

**Figure 3:**
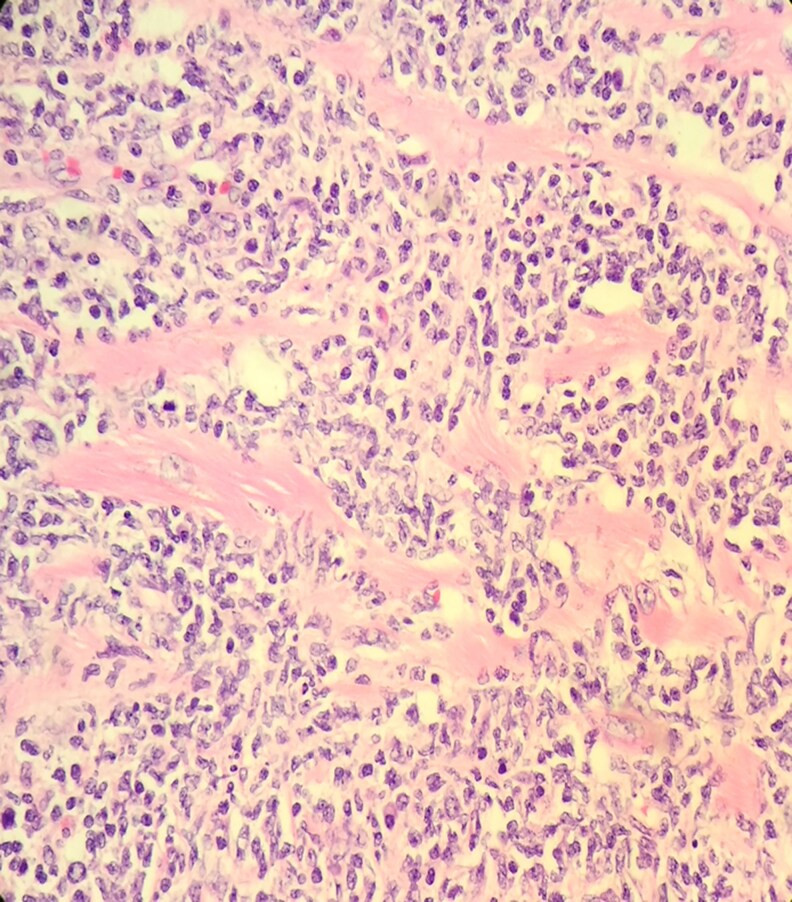
Pathological analysis of the explanted heart shows a massive infiltration of lymphocytes associated with follicular lymphoma pattern and massive myocardial necrosis.

**Figure 4:**
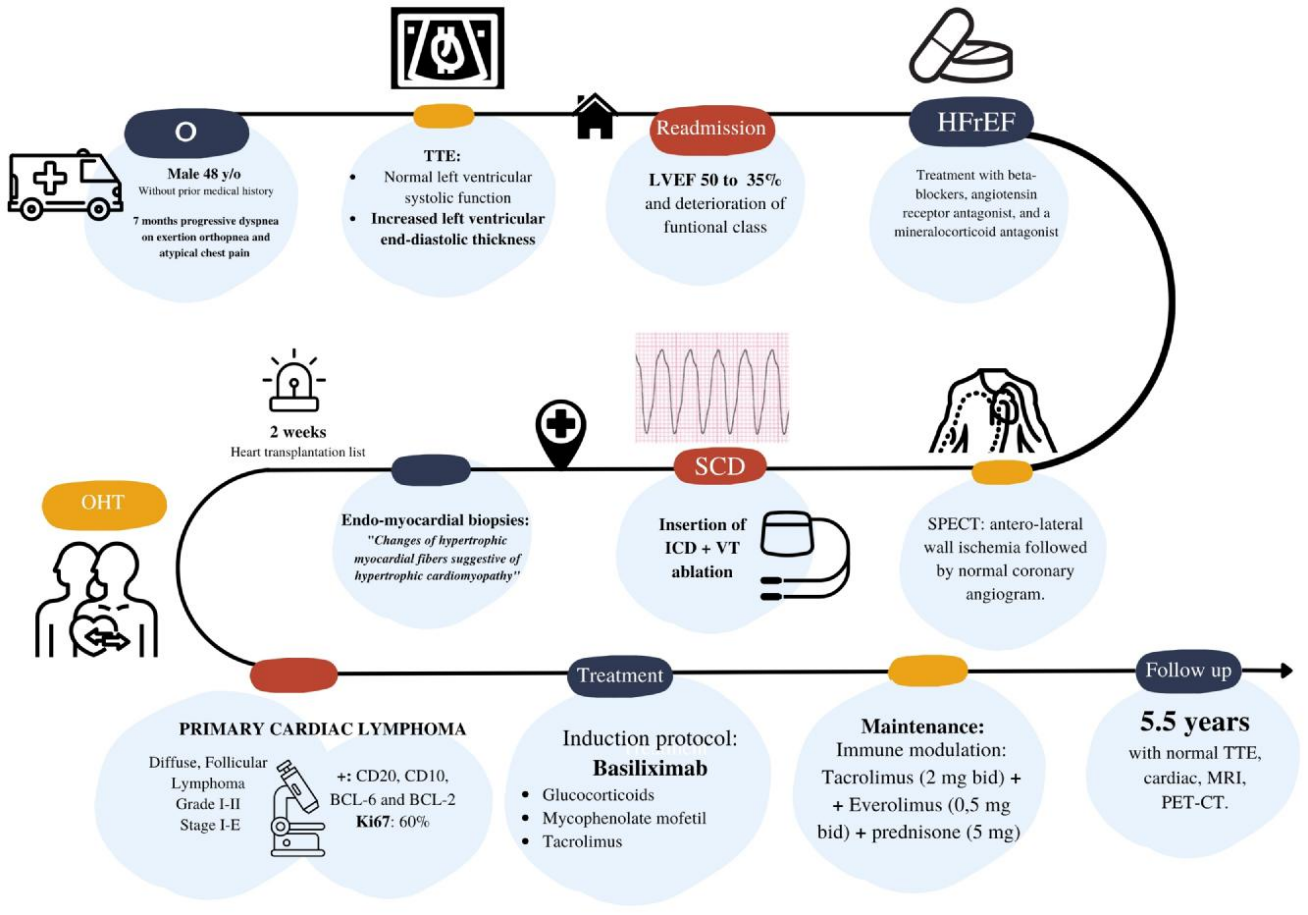
Timeline the most representative moments of the case. TTE, trans-thoracic echocardiogram; HFpEF, heart failure with preserved ejection fraction; LVEF, left-ventricular ejection fraction; SPECT, single-photon emission computed tomography; SCD, sudden cardiac death; ICD, implantable cardiac defibrillator; VT, ventricular tachycardia; OHT, orthotopic heart transplantation; MRI, magnetic resonance imaging; PET-CT, positron emission tomography.

Four days after transplantation, the pathological analysis of the explanted heart showed a PCL with significant lymphocyte infiltration, myocardial necrosis, and interstitial oedema. (*Figure 3*). Immuno-phenotypic analysis demonstrated the neoplasm was composed of B cells with expression of CD20, CD10, BCL-6, and BCL-2. CD5 and Cyclin D1 were not expressed and Ki67 showed a proliferative index of 60%. These results were indicative of a diffuse, Follicular Lymphoma, grade I–II, stage I–E, with extensive cardiac involvement (*Figure 3*). There was no evidence of extra-cardiac extension after bone marrow biopsy and full-body PET-CT scan. Of note, the resection edges of the specimen were negative for tumour. If an additional extension of the lymphoma had been noticed after the transplant, probably the same regimen of chemotherapy would have been utilized but, with a longer duration and his prognosis would have worsened significantly. The chronological record of the most important events of the case is shown in *Figure 4*.

To avoid the cardiotoxic effects of the R-CHOP scheme in the setting of a Stage I PCL-follicular non-Hodgkin lymphoma and, with a very recent heart transplant with complete excision of the tumour, Rituximab + Bendamustine were initiated.^4–7^ He received two initial combined cycles, followed by rituximab monotherapy for four semester cycles, obtaining a complete metabolic response on follow-up including normal PET-CT, beta 2 microglobulin levels, and bone marrow biopsy. There was no evidence of cardiotoxicity Rituximab, such as cardiomyopathy, coronary spasm and Takotsubo syndrome.^8^ The serial TTEs after OHT have demonstrated a normal graft function, and three consecutive biopsies were negative for cellular and humoral rejection.

After a scheduled heart biopsy is done 2 months after transplant for rejection evaluation, the patient reported marked dyspnoea and a new TTE showed flail of the anterior leaflet of the tricuspid valve due to the rupture of a tendinous cord, resulting in torrential tricuspid regurgitation (TR). Eventually, the patient underwent a repair with a ring and neo-chords but was not successful, and for that reason, the decision was made to perform tricuspid valve replacement with a bio-prosthesis, after ruling out infective endocarditis, another significant risk factor.

After 5.5 years, the patient remains asymptomatic, with adequate function of the graft and with a NYHA FC I. No further right ventricle biopsies have been performed due to the complication noted and a cardiac magnetic resonance imaging (MRI) has been performed periodically without findings suggestive of rejection or tumour recurrence. The decision to use a non-cardiotoxic chemotherapy scheme was driven by the need to maintain the therapy over time and because rituximab plus bendamustine have shown at least similar efficacy to the standard R-CHOP regimen with a much better toxicity profile.^6,7^

The authors ensure this case report study has been carried out according to the ethical guidelines outlined by The Transplantation Society, including the Declaration of Helsinki, the Declaration of Istanbul, and the ethical principles outlined by the Committee on Publication Ethics. Due to the kind of our case report, the study was exempt from approval from an institutional ethics board.

## Discussion

PCL is rare and usually involves the heart and the pericardium only, without extra-cardiac extension. It constitutes only 1% of primary cardiac tumours and 0.5% of extra-nodal lymphomas and its prognosis is usually poor with a mean survival of 12 months.^2,9–12^ Due to the rare diagnosis, consensus guidelines for workup and treatment are lacking. The initial presentation of a possible rapidly deteriorating hypertrophic cardiomyopathy made the final diagnosis more challenging. In addition, the early implantation of a non-conditional MRI cardioverter limited the opportunity to use multimodality. The rapid and progressive deterioration of ventricular function with VT-S in the setting of a presumptive diagnosis of hypertrophic cardiomyopathy were the main reasons for listing him as 1A status for heart transplant. Trans-venous biopsy guided by images has been useful for the diagnosis of PCL but may be not diagnostic in some patients,^1^ including the current case. The role of heart transplantation in patients with malignant cardiac tumours is not clearly established and, for that reason, the International Society for Heart and Lung Transplant (ISHLT) guidelines section recommends a close multidisciplinary approach including Oncology consultation to stratify the risk of tumour recurrence after heart transplantation. This evaluation was not done in this case granted the urgency of the clinical status and because PCL was not considered in the initial differential diagnosis. Although the tumour was resected completely with negative surgical margins and no apparent extra-cardiac involvement was noted, continuous follow-up with our Oncology colleagues is mandatory in our patients. TR is a complication that has been reported in cohort studies in up to 1% to 61,5%. No associations have been reported either with the number of endomyocardial biopsies performed or with episodes of acute rejection, immunosuppressive or chemotherapy regimens^13^ and the incidence of TR has had a downward trend over time with some modifications in the technique. The choice of immunosuppression in this case, with an unknown diagnosis of PCL, made us consider the risk of recurrence, acknowledging heart transplant patients have an increased risk of developing cancer, particularly post-transplant lymphoproliferative disease (PTLD).^14^ Associated factors recognized in PTLD are immunosuppressive therapy, specifically the use of cyclosporine and azathioprine, as well as the use of polyclonal or monoclonal anti-lymphocyte antibodies and Epstein-Barr virus (EBV) infection.^15^ Taking these factors into account, we decided to use m-TOR, whose effect on the inhibition of the proliferation of T and B lymphocytes has advantages over the usual regimen due to its antiproliferative effect in graft vascular disease^11^ and neoplasms,^12^ combined with low doses of tacrolimus in order to prevent rejection in a patient with difficulty in follow-up with biopsies. The scheme of low doses of tacrolimus (2 mg bid) + Everolimus (0,5 mg bid) and prednisone (5 mg) withdrawn after one year of transplantation resembles the case described by Ried *et al.*^1^ Of note, we have modified the doses of immunosuppressant to lower levels considering the additional cycles of chemotherapy with rituximab and trying to avoid infectious complications or bone marrow toxicity.

This case highlights the importance of suspecting PCL in patients presenting with rapidly deteriorating hypertrophic myocardiopathy associated with refractory ventricular and supraventricular arrhythmias. In the absence of a PCL ‘goal-standard’ therapy, alternative schemes to R-CHOP may be effective and safe. In addition, modified immunosuppression can be a valid alternative to avoid associated toxicity. We also highlight the safety of a long-term, non-invasive surveillance for graft rejection or lymphoma relapse. Active cancer may be reconsidered as a contraindication for heart transplantation in selected patients. International-level registries may contribute to extending the knowledge in this uncertain area.

## Conclusion

The optimal treatment of PCL remains a challenge and it’s not clear if transplantation plus chemotherapy is an option for selected stage I cases. In this patient, the management of combined immunosuppression with chemotherapy was unpredictable. Also, his long-term follow-up has been restricted to clinical and imaging findings (TTE and MRI every six months during the first year and annually thereafter) granted periodic biopsies are prohibited due to the risk of damage to the bio-prosthesis. Fortunately, during his follow-up, he did not show signs of rejection. Yearly workup including PET-CT, and routine blood workup including beta microglobulin, CBC, LDH, renal and hepatic function profiles remain unremarkable.

## Data Availability

The data underlying this article will be shared on reasonable request to the corresponding author.

## Lead author biography

At Fundación Cardioinfantil - LaCardio, I am a cardiologist dedicated to the complexities of heart failure and cardiometabolic disorders. My passion extends to clinical research, where I strive to translate innovative findings into improved patient outcomes.

## References

[1] Ried M, Rupprecht L, Hirt S, Zausig Y, Grube M, Resch M, et al Sequential therapy of primary cardiac lymphoma with cardiectomy, total artificial heart support, and cardiac transplantation. J Hear Lung Transplant 2010;29:707–709. 10.1016/j.healun.2010.01.014 20227299

[2] Zhuang S, Chang L, Feng X, Hu W, Yang Z, Zhang Y. Primary cardiac lymphoma: a clinicopathological study of 121 cases. Front Oncol 2025;14:1509100. 39839800 10.3389/fonc.2024.1509100 PMC11746027

[3] Asadian S, Rezaeian N, Hosseini L, Toloueitabar Y, Komasi MMH. The role of cardiac CT and MRI in the diagnosis and management of primary cardiac lymphoma: a comprehensive review. Trends Cardiovasc Med 2022;32:408–420. 34454052 10.1016/j.tcm.2021.08.010

[4] Fujita Y, Ikebuchi M, Tarui S, Irie H. Successful combined treatment of primary cardiac malignant lymphoma with urgent cardiac operation and chemotherapy. Circ J 2009;73:967–969. 19088397 10.1253/circj.cj-08-0064

[5] Fernández-Martínez J, Descalzo-Buey M, Menduiña-Gallego I, Pujadas-Olano S, Viladés-Medel D, Salido-Iniesta M, et al Regression of cardiac lymphoma with chemotherapy. JACC Case Rep 2023;29:102166. 38264308 10.1016/j.jaccas.2023.102166 PMC10801802

[6] Rummel MJ, Niederle N, Maschmeyer G, Banat GA, Von Grünhagen U, Losem C, et al Bendamustine plus rituximab versus CHOP plus rituximab as first-line treatment for patients with indolent and mantle-cell lymphomas: an open-label, multicentre, randomised, phase 3 non-inferiority trial. Lancet 2013;381:1203–1210. 23433739 10.1016/S0140-6736(12)61763-2

[7] Hitz F, Zucca E, Pabst T, Fischer N, Cairoli A, Samaras P, et al Rituximab, bendamustine and lenalidomide in patients with aggressive B-cell lymphoma not eligible for anthracycline-based therapy or intensive salvage chemotherapy—SAKK 38/08. Br J Haematol 2016;174:255–263. 27018242 10.1111/bjh.14049

[8] Ng KH, Dearden C, Gruber P. Rituximab-induced takotsubo syndrome: more cardiotoxic than it appears? BMJ Case Rep 2015;2015:1–4. 10.1136/bcr-2014-208203 PMC4369036 25733089

[9] Motwani M, Kidambi A, Herzog BA, Uddin A, Greenwood JP, Plein S. MR imaging of cardiac tumors and masses : a review of methods. Radiographics 2013;268:26–46. 10.1148/radiol.13121239 23793590

[10] Yin K, Brydges H, Lawrence KW, Wei Y, Karlson KJ, McAneny DB, et al Primary cardiac lymphoma. J Thorac Cardiovasc Surg 2022;164:573–580.e1. 33158567 10.1016/j.jtcvs.2020.09.102

[11] Eisen HJ, Kobashigawa J, Starling RC, Pauly DF, Kfoury A, Ross H, et al Everolimus versus mycophenolate mofetil in heart transplantation: a randomized, multicenter trial. Am J Transplant 2013;13:1203–1216. 23433101 10.1111/ajt.12181

[12] Furukawa S, Wei L, Krams SM, Esquivel CO, Martinez OM. PI3Kδ inhibition augments the efficacy of rapamycin in suppressing proliferation of Epstein-Barr virus (EBV)+ B cell lymphomas. Am J Transplant 2013;13:2035–2043. 23841834 10.1111/ajt.12328 PMC4076428

[13] Fiorelli AI, Benvenuti L, Aielo V, Coelho AQ, Palazzo JF, Rossener R, et al Comparative analysis of the complications of 5347 endomyocardial biopsies applied to patients after heart transplantation and with cardiomyopathies: a single-center study. Transplant Proc 2012;44:2473–2478. 23026623 10.1016/j.transproceed.2012.07.023

[14] Mudigonda P, Berardi C, Chetram V, Barac A, Cheng R. Implications of cancer prior to and after heart transplantation. Heart 2022;108:414–421. 34210749 10.1136/heartjnl-2020-318139

[15] Li J, Liu Q, Peng Q, Dong S. Diagnosis of rapidly progressed primary cardiac lymphoma in liver transplant recipient: a case report. Front Oncol. 2022;12:1014371. 36212392 10.3389/fonc.2022.1014371 PMC9544802

